# Natural language processing reveals differences in mental time travel at higher levels of self-efficacy

**DOI:** 10.1038/s41598-024-76959-w

**Published:** 2024-10-25

**Authors:** Laurin Plank, Armin Zlomuzica

**Affiliations:** Department of Behavioral and Clinical Neuroscience, Ruhr-University Bochum (RUB), Massenbergstraße 9-13, 44787 Bochum, Germany

**Keywords:** Mental time travel, Episodic memory, Episodic future thinking, Self-efficacy, Natural language processing, Psychology, Human behaviour

## Abstract

The (re-)construction of past and future personal experiences, termed mental time travel (MTT), is highly adaptive and contributes to self-related beliefs and attitudes. Mounting evidence suggests that self-efficacy (SE), the belief that one can overcome obstacles on their own account, is functionally related to MTT. In the present study, we used novel methods for the analysis of MTT narratives based on natural language processing (NLP) to investigate the relation between SE and MTT. We demonstrated that self-efficacious participants remembered and imagined experiences which were semantically less similar and contained a wider variety of contents. Additionally, increased SE was related to a positive reappraisal of personal episodes and reports of a more active role within mental scenarios. In conclusion, NLP appears to be a valuable method to quantify changes to the (re-)construction of personal experience that might support cognitive and emotional functioning.

## Introduction

Mental time travel (MTT) refers to the ability to remember and imagine experiences in one’s personal past and future^1–3^. MTT is highly adaptive as it allows to flexibly adapt behavior to dynamic and complex environments^4^. MTT is thought to enable future planning based on past experience (directive-function), navigation of social environments (social-function) and contributes to a stable and continuous sense of self (self-function)^5–7^. Due to its widespread relevance, MTT has often been considered in the context of mental health. An impoverished ability to mentally traverse time has a detrimental impact on the onset and progression of various mental disorders, in particular anxiety disorders^8,9^, trauma-related disorders^8,10,11^ and depression^8,12^.

The (re-)construction of personal experiences has been suggested to be dependent upon self-related processes such as attitudes, goals and beliefs^13^. One aspect of the self which has been shown to modulate the retrieval of autobiographical memories is the belief of self-efficacy (SE)^14–16^. SE is defined as the belief that one carries the necessary means to overcome encountered challenges^17^. An experimental increase of SE by means of verbal feedback has been shown to increase the specificity of autobiographical recall and enhance social problem solving^14,16^. Furthermore, increased SE results in MTT narratives that are marked by more positive words and SE statements^16^. Following exposure to a trauma analog, subjects whose SE was experimentally increased experience fewer involuntary memory intrusions^18^, suggesting that SE appears to influence the flexibility of memory recall. In a similar vein, SE tends to correlate negatively with inflexible cognitive styles such as rumination and worry^19–22^. Finally, SE was found to positively correlate with the perceived autobiographical coherence^23^. The authors suggested that it is the perception of meaningful connections between past experiences which fosters SE beliefs and creates a sense of personal agency^23^.

Prior research on MTT and SE has exclusively relied on subjective report^19–23^ and manual scoring of autobiographical narratives^14–16^. Although these methods have yielded various important insights, they are constrained in two main ways. Firstly, self-report is subject to biases, some of which are especially pronounced in the assessment of internal states^24–26^. Secondly, there are certain textual features, that may not be quantifiable by human raters^27^. One domain of research that falls into this category is the content (semantic) analysis of autobiographical memory narratives. There is an almost endless number of possible dimensions upon which the semantics of a text might be scored^28^. As a consequence, the content of memories has largely been ignored^29^. The inherent complexity of this task has recently prompted researchers to apply data-driven computational models to the study of text content^28^. For example, in a study by Sheldon and colleagues, participants generated memory narratives, which were then subjected to content analysis using Latent Dirichlet Allocation (LDA) and semantic embedding methods. The authors could show that older adults tended to generate autobiographical memories that span a wider variety of topics. Additionally, it was found that memory narratives of younger adults exhibit a higher level of coherence^29^. Vrana and colleagues^30^ studied the coherence of trauma and neutral narratives and found that trauma narratives tended to be less coherent. Additionally, over multiple writing sessions, the coherence of the trauma narratives increased, suggesting that a low level of narrative coherence was indicative of a poor organization of memories^30^. In another important study, Lee and Chen^27^ generated semantic networks of memory narratives and could show that central events, i.e. those which are closely linked to many other events, are better remembered. In summary, novel insights are being generated by applying NLP models to the study of memory narratives. While these methods have been used in the context of research on aging^29^, memory performance^27^ and trauma^30,31^, they have not yet been employed to study the relation between MTT and self-related beliefs, such as SE.

To summarize, prior evidence suggests a tight link between MTT and SE. Addressing the concerns outlined above might reveal mechanism behind the positive effects of SE on mental health that have not been considered thus far^32–37^. Consequently the goal of the present study was twofold. Based on previous research on SE and MTT, we first constructed textual features which we hypothesized to be relevant to SE. Using experimental and machine-learning (ML) based methods, the features were then validated. Following validation, we were interested in whether natural variations in trait SE might be related to these features. Unlike previous studies^14,15,18,38^, we did not induce SE experimentally, but chose to examine trait variations in SE.

Several NLP-derived textual features were used, namely the emotional tone, narrative coherence, future-past similarity and MTT flexibility (see the subsection “Textual analysis” in the methods section for a technical description). The emotional tone was extracted via sentiment analysis^39^ and represents an objective measure of the emotionality of a MTT narrative. It was previously shown that a SE induction causes subjects to use a higher frequency of positive words to describe their MTT^14^. However, in this earlier study, the emotionality of narratives was assessed by counting the frequency of positive and negative emotion words, which ignores contextual semantics. In contrast, sentiment analysis is sensitive to negations (e.g., *not* amused) and intensifiers (e.g., *extremely* angry). We hypothesized that high SE would coincide with more positive MTT narratives. MTT flexibility was used as a measure of perseverative/inflexible cognition such as worry and rumination, which has been shown to be lower in highly self-efficacious subjects^19–22^. By crafting a computational/experimental measure, we quantify the cognitive style itself, rather than relying on self-report as was previously done^19–22^. We expected highly self-efficacious subjects to show higher MTT flexibility. Coherent autobiographical narration has been related to various beneficial consequences^40,41^ and narrative coherence might support SE beliefs^23^. Additionally, the fact that a SE efficacy induction reduces the frequency of intrusions induced by a traumatic film^18^ might indicate that SE beliefs support proper memory integration and organization. This memory (dis)organization can be quantified using computational methods^29,30^. We hypothesized a higher level of memory coherence for subjects with higher SE. The thematic similarity between past and future events has been investigated in the context of Alzheimer’s disease (AD). It was found that patients with AD were less able to imagine future event whose content differed from past events^42^, indicating that these patients tend to perseverate on the content of prior memory recall. In the present study, a related computational measure was constructed to test whether this perseveration would be decreased at higher levels of SE, which we expected. This would indicate that more self-efficacious people are better able to imagine future episodes that are distinct from past ones^17^. In addition to the textual features, we explored the phenomenology associated with MTT in self-efficacious subjects. The phenomenological dimensions chosen were based on autobiographical memory research in depression^43,44^. We expected a more positive subjective emotional valence for subjects with higher SE.

## Methods

### Participants and demographic data

The experiment was performed online using Qualtrics, a web-based survey tool. Participants were recruited via the university’s research study website. Exclusion criteria were a mental or neurological disorder, current psychotherapy, prescription-only medication, substance abuse, and insufficient knowledge of the German language. All experimental procedures were approved by the university’s local ethics committee (approval #857) and the study was performed in accordance with the Declaration of Helsinki. All participants provided informed consent and were compensated for their participation either with student course credit points or a 15€ gift card for a global retailer.

We determined that to achieve a statistical power of 0.8 in discovering a medium-sized effect (r = 0.3) in the correlational analyses, a minimum sample size of 84 was necessary (G*POWER^45^). 108 participants were recruited because we expected some exclusion in the text analyses due to poor data quality. Participants were predominantly female (67.6%) with a mean age of 25.8 years (SD = 6.45, range = 18–54). Most participants reported either A-levels (45.37%) or a Bachelor’s degree (37.96%) as their highest achieved level of education. The sample had low depression-, anxiety- and stress levels with 11.11%, 17.59% and 18.52% of participants having DASS-21 sub scores above the cutoff for the dimensions depression, anxiety and stress, respectively.

### Experimental procedures

#### Mental time travel test (MTT-T)

The MTT test (MTT-T) was modelled after previously employed tasks such as the autobiographical memory test (AMT)^46^ and the episodic future thinking test (EFT-T)^43^. Before the start of the MTT-T, participants received detailed instructions to only report events (1) which happened or could happen in the past or future, (2) which they were personally involved in, (3) which lasted less than 24 h, and (4) which took place at a specific place at a specific time. The participants were asked to report their MTTs with as much detail as possible and to reference their feelings, thoughts, and emotions. Two examples for cue word prompted MTT-narratives (one specific and detailed example and one unspecific and undetailed example) served as part of the instruction. After the general introduction and instruction the MTT-T started.

The MTT-T consisted of 12 trials in total. In each trial, participants were presented with two cue words. One cue word specified the temporal orientation of the MTT (future or past), while the other served as an inspiration as to the content of the MTT. The content cue word had either a negative, neutral, or positive emotional valence. There were a total of six content cue words (two for each valence). Each of the content cue words was presented twice, once in combination with the word “future” and once with the word “past”. The order of the trial presentations was randomized. The selection process for the content cue words is described in the supplementary information section “Cue word selection”, including Table S1.

The exact procedure of a trial went on as follows: After participants were presented with a cue word pair and thought of an event to be reported, they were asked to write a text describing this particular event in as much detail as possible (time limit 180 s). Participants were asked to report a different event in each trial. Thereafter, participants were asked to rate the phenomenological characteristics of the reported event. The participants rated the event on 9 visual analogue scales assessing the vividness (1 = not at all – 7 = very), emotional arousal (1 = not at all – 7 = very), emotional valence (1 = very negative – 7 = very positive), detailedness (1 = not at all – 7 = very), personal relevance (1 = not at all – 7 = very), use of mental imagery (1 = not at all – 7 = very much), perspective (1 = observer/third person – 11 = own eyes/first person), level of activity (1 = completely passive – 7 = completely active), and the dynamic vs. static nature of the scene (1 = completely static – 7 = completely dynamic). The temporal distance from the present was assessed using a Likert scale (1 = within 1 day; 2 = within 1 week; 3 = within 1 month; 4 = within 1 year; 5 = over 1 year; 6 = over 10 years).

### Questionnaires

After the MTT-T, participants were asked to complete questionnaires assessing demographic data, SE expectations^47^, and depression-, anxiety- and stress symptoms^48,49^. The SE questionnaire yielded an unstandardized Cronbach’s α of 0.89, indicating good internal consistency^47^.

### Textual analysis

#### Text length and emotionality

The narratives’ length and emotionality were derived using the “textblob_de” package in python. To this end, narratives were tokenized (divided into words) and a sentiment analysis was conducted. The sentiment analysis yields a polarity measure ranging from -1 (negative) to 1 (positive). If sentiment analysis may accurately capture the emotionality of the produced narratives, valence ratings should be the highest in positively cued trials, followed by neutrally cued and negatively cued trials. This validation is provided in the results section under the subheading “Emotionality”.

#### Validation of semantic vector embeddings

**Fig. 1:**
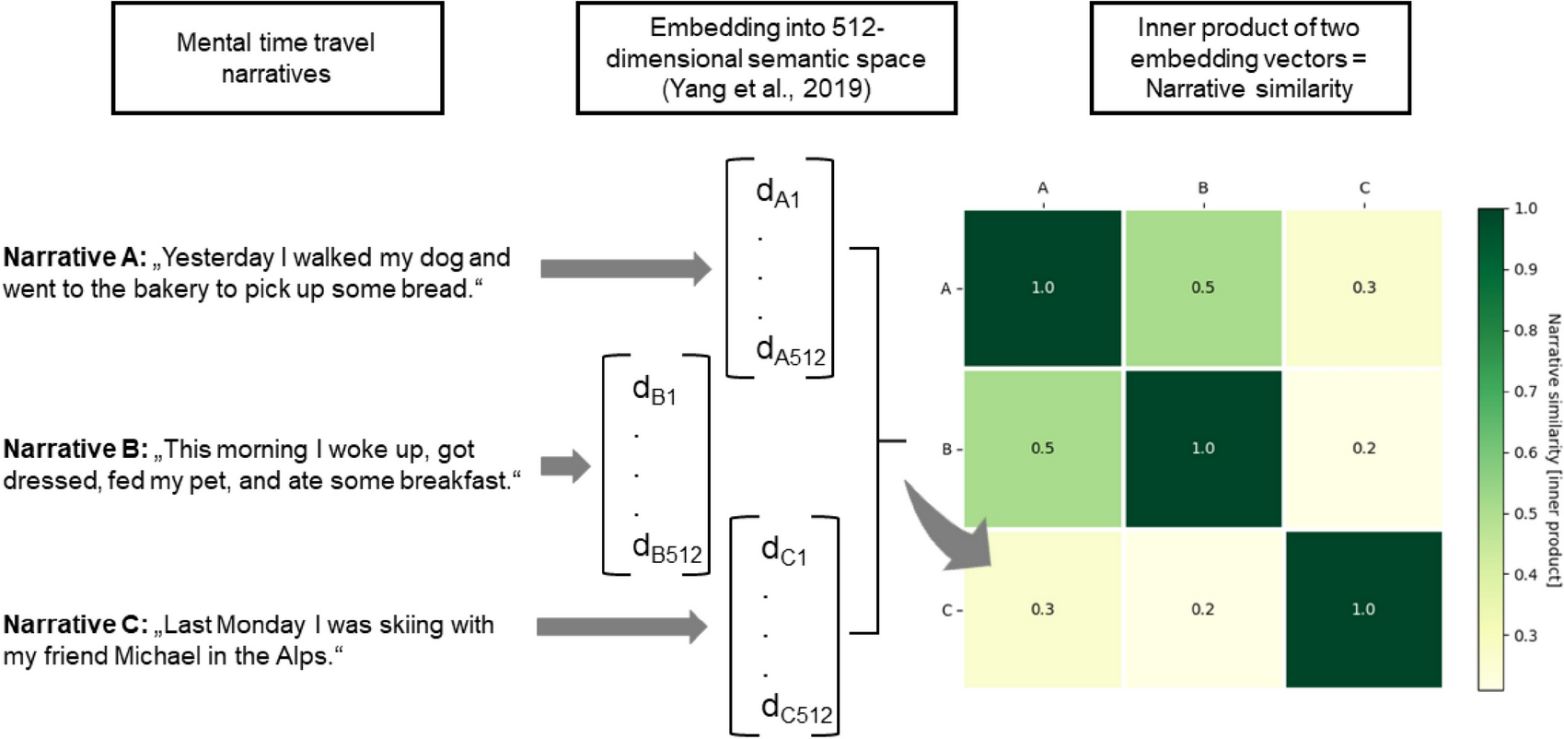
Semantic analysis of memory narratives using a sentence embedding model^50^. First, textual narratives are encoded into 512-dimensional space using the multilingual Google Universal Sentence Encoder^50^. The inner product between two embedding vectors indicates the degree of semantic similarity between two narratives. A inner product of one indicates identical narratives. Lower values indicate a lower degree of narrative similarity. A narrative similarity matrix is shown for three exemplary narratives. The narratives are fictional and were not produced by the participants of this study. Values are rounded to the first digit. Narrative A and B show a higher degree of similarity than narrative A and C (or B and C). This makes intuitive sense given that narrative A and B share more topics (pet and food) than narrative A and C (or B and C).

The “multilingual Google universal sentence encoder” (mGUSE)^50^ was used to quantify the semantics of a given MTT report. The mGUSE transforms texts into a 512-dimensional vector, which represents their meaning or content (semantics). The inner product of two text embeddings indicates the degree of semantic overlap between them^29,31^. Figure 1 displays the process of extracting the semantic similarity of exemplary MTT narratives.

**Fig. 2:**
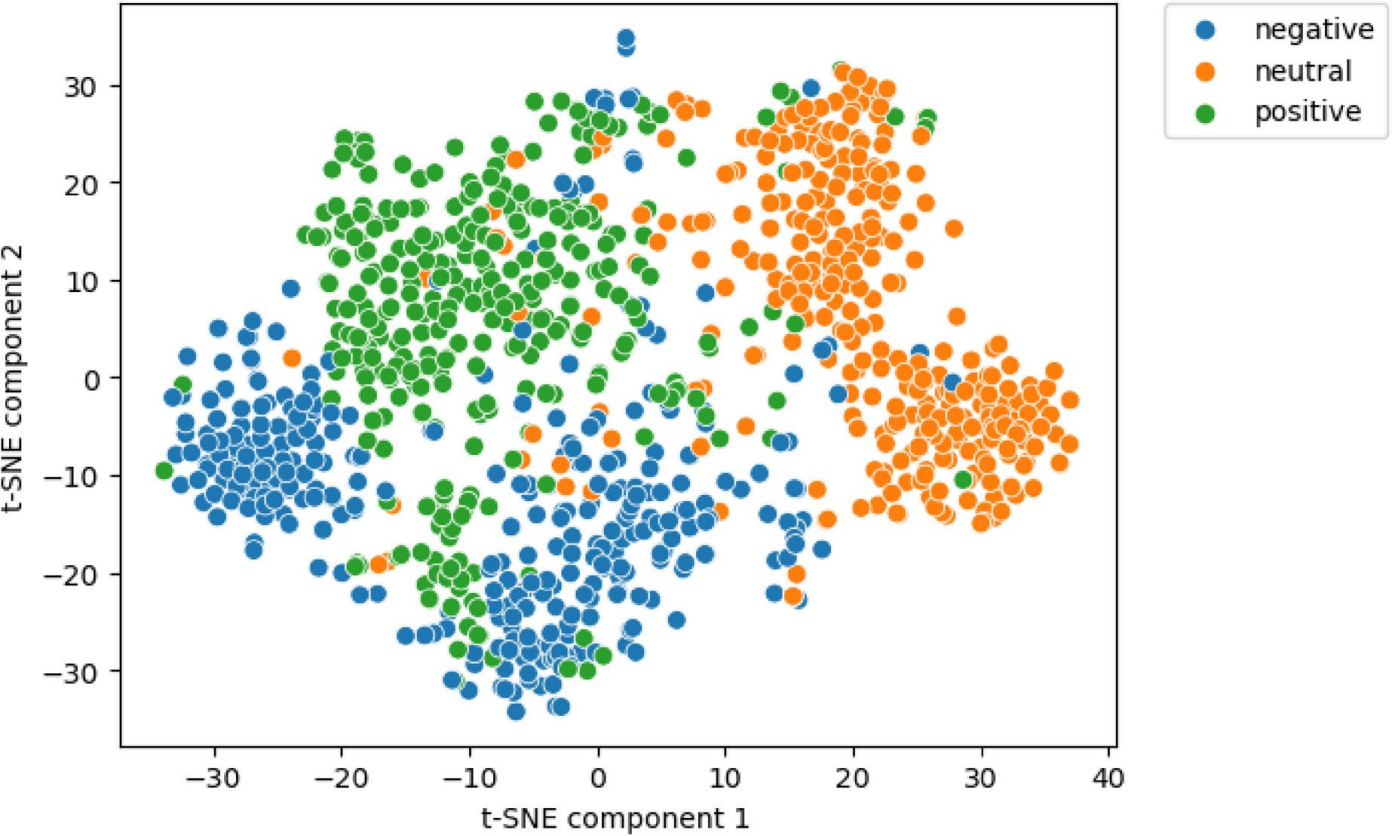
Projection of mental time travel (MTT) narrative embeddings across all participants into two-dimensional space via t-distributed stochastic neighbor embedding (t-SNE)*.* Each point corresponds to the projection of a mental time travel narrative (MTT) embedding into two-dimensional space. Projection from 512- to two-dimensional space was achieved using t-SNE^51^. Briefly, t-SNE is a nonlinear dimensionality reduction technique, which attempts to cluster points in low dimensional space if they are also clustered in high-dimensional space. t-SNE was computed using the scikit-learn package in python using the default parameters. The plot includes all 984 narratives produced by the participants. In addition, narrative embeddings are color coded according to the emotional valence of the cue word which prompted the MTTs. Narrative embeddings appear to form clusters depending on the cue words’ emotional valence. MTTs prompted by a given cue word valence appear more similar to each other than to MTTs prompted by another cue word valence. As t-SNE only retains the data’s local, but not global structure, the distance between clusters should not be interpreted.

To further test whether the mGUSE successfully encodes the narratives’ semantics, we projected all 984 MTT narratives produced by the participants from 512- into two-dimensional space via t-distributed stochastic neighbor embedding (t-SNE)^51^. t-SNE is a nonlinear dimensionality reduction technique, which attempts to cluster points in low dimensional space if they are also clustered in high-dimensional space. In Fig. 2, narrative embeddings are color coded according to the emotional valence of the cue word which prompted the MTT. A clear clustering pattern based on the cue word valence emerged. MTTs prompted by a given cue word valence appear more similar to each other than to MTTs prompted by another cue word valence. It follows that the mGUSE successfully encodes the meaning of MTT narratives and that participants tended to adhere to content cueing in the MTT-T.

#### Semantic feature extraction

To determine the degree of similarity between MTT-narratives into the past and future^42^ stimulated by the same cue word, we calculated the inner product of the corresponding vectors, hereafter referred to as future-past similarity. By averaging variances across all 512 embedding dimensions over multiple MTT-narratives, we determined the degree of semantic spread for the entire set or a subset of MTT-narratives. We refer to this measure as semantic variance. Subjects who report widely different MTTs will score more highly on this measure than subjects who tend to report similar MTTs. Thus, semantic variance serves as a measure of MTT flexibility. We also calculated the average inner product of the embeddings of consecutive sentences within a MTT-narrative as a measure of the coherence of the narrative. MTT narratives in which consecutive sentences are more similar on a semantic level may be considered more coherent. In the following, this measure will be referred to as narrative coherence^29,30,52^. The computational quantification of narrative coherence followed the approach by Sheldon et al.^29^. A summary of all extracted textual features is given in Table 1.

**Table 1:** Description of extracted textual features.

Term	Method	Interpretation
Emotional tone	Sentiment analysis (“textblob_de”)	Emotional valence of a MTT narrative
Future-past similarity	Inner product of semantic vector embeddings of cue word congruent narratives (mGUSE)	Content similarity between narratives of MTTs into the past and future prompted by the same cue word
Semantic variance	Average variance over all embedding dimensions over a set of vector embeddings (mGUSE)	Content diversity over a set of MTT narratives – MTT flexibility
Narrative coherence	Average inner product of vector embeddings of subsequent sentences within a narrative (“textblob_de”, mGUSE)	Coherence in the content development of a narrative

Tokenization refers to the process of dividing a body of text into smaller units, in this case words. *mGUSE* multilingual Google Universal Sentence Encoder^50^.

### Statistical analysis

For most tests, mixed 3-way analyses of covariance (ANCOVA, type III) with within factors *Directedness* (future vs. past) and *Valence* (negative vs. neutral vs. past) and covariate *Self-efficacy* were employed. The use of a different statistical test is explicitly stated. Because semantic variance is a spatial measure reflecting the relationship between trials, not an aggregate of trial-level scores, ANCOVAs could not be used in this particular case. Here, correlation tests were simply used instead. Assumptions of sphericity were tested for using Mauchly’s test for sphericity and when violated, the Greenhouse–Geisser method was used to correct p-values and the degrees of freedom. Post-hoc pairwise comparisons were calculated using two-sided t-tests. As a post-hoc test for the influence of the covariate *Self-efficacy*, Pearson correlations were used. Post-hoc correlations between SE and the mean dependent variable values across all trials were always calculated. Post-hoc tests for SE interactions were only calculated when the ANCOVA yielded significance. Multiple comparisons were adjusted for using Bonferroni-Holm’s method. Statistical tests were considered significant at p-values below 0.05. Positive results regarding the factors *Directedness* and *Valence*, as well as descriptive statistics for all dependent variables are reported in the supplementary information section “Supplementary results”, including Tables S2 and S3.

### Software

Textual analyses and visualizations were performed in python (version 3.9.18), including the packages “numpy” (1.26.2), “pandas” (2.1.1), “tensorflow_hub” (0.15.0), “scikit-learn” (1.4.1), “seaborn” (0.12.2), and “textblob_de” (0.4.3). Statistical analysis and plot creation was performed in R (4.3.1), including the packages “readxl” (1.4.3), “rstatix” (0.7.2), “effsize” (0.8.1), “ggplot2” (3.4.3), “tidyr” (1.3.0), “car” (3.1–2), “ltm” (1.2–0), and “stats” (4.3.1). Microsoft PowerPoint was used for plot creation.

## Results

### Data exclusion

The sentence embedding models require texts to be at least one sentence long^50^. Because sentences can be only a couple words long, we decided to set the cutoff for the minimal text length at 20 words. Out of the 108 participants, 82 (75.93%) responded with at least 20 words for each MTT. Narratives were manually screened for any nonsense content and none had to be excluded on that basis. In tests involving the narrative coherence measure, we only considered subjects who reported at least two sentences on each narrative, as otherwise the coherence scores cannot be calculated.

### Wordcount

Participants’ MTT narratives had a mean wordcount of 71.51 [SD = 22.88, range = 31.58–134.5] across all trials. There was no significant correlation between SE and the average wordcount of the narratives across all trials, r(106) = − 0.0002, p = 1. As SE did not correlate with the text length, the wordcount was not included as a covariate in subsequent analyses.

### Emotionality

We first report results for the within factor *Valence* to validate whether the narratives’ emotional valence could be captured via sentiment analysis. There was a significant effect of the factor *Valence*, F(2,120) = 52.43, p < 0.001, η^2^p = 0.47. Positively cued narratives [M = 0.29, SD = 0.15] were significantly more positive than neutrally cued [M = 0.08, SD = 0.12], t(153.08) = 9.91, adj. p < 0.001, d = 1.55, and negatively cued [M = − 0.01, SD = 0.17] narratives, t(160.68) = 12.09, adj. p < 0.001, d = 1.89. Neutrally cued narratives [M = 0.08, SD = 0.12] were significantly more positive than negatively cued narratives [M = − 0.01, SD = 0.17], t(146.52) = 3.98, adj. p < 0.001, d = 0.62. Additionally, there was a significant positive correlation between the objective and subjective emotional valence of an episode, r(982) = 0.44, p < 0.001. Altogether, these results suggest that the sentiment analysis was indeed successful in extracting the narratives’ emotional valence.

There was a significant main effect of the covariate *Self-efficacy* on the subjective emotional valence, F(23,84) = 2.12, p = 0.007, η^2^p = 0.37. SE showed a significant positive correlation with the mean subjective valence across all trials, r(106) = 0.32, adj. p = 0.007. In contrast, SE did not correlate with the average objective emotional valence of the narratives across all trials, r(80) = 0.01, p = 1. The fact that SE correlated positively with the subjective, but not objective emotional valence might indicate an emotional reappraisal of personal episodes. We directly tested this possibility by first transforming the subjective valence ratings into the same range as the objective valence ratings (− 1 to 1). We then calculated the difference between the subjective and objective emotional valence and computed another ANCOVA. There was no significant main effect of the covariate *Self-efficacy*, F(21,60) = 1.47, p = 0.13, η^2^p = 0.34. SE was, however, significantly correlated with the average valence difference score in the post-hoc test, r(80) = 0.24, p = 0.033. Higher levels of SE coincided with the evaluation of experiences as significantly more positive relative to the emotional valence of written narratives.

### Phenomenology

There was a significant main effect of the covariate *Self-efficacy* on the subjective level of activity (whether subjects reported an active or passive role), F(23,84) = 2.08, p = 0.008, η^2^p = 0.36. SE and the subjective level of activity across all trials showed a significant positive correlation, r(106) = 0.27, adj. p = 0.045. There was a significant interaction between the covariate *Self-efficacy* and the factor *Directedness* on the vividness, F(23,84) = 1.81, p = 0.027, η^2^p = 0.33. Upon correction for multiple testing, this effect seized to be significant in the post-hoc correlation, r(106) = 0.23, adj. p = 0.13.

### Narrative coherence

The covariate *Self-efficacy* did not show a significant main effect on coherence scores, F(20,47) = 0.65, p = 0.9, η^2^p = 0.22. Post-hoc testing also revealed no significant correlation between SE and the average coherence across all trials, r(66) = − 0.6, p = 0.5.

### Semantic variance

**Fig. 3:**
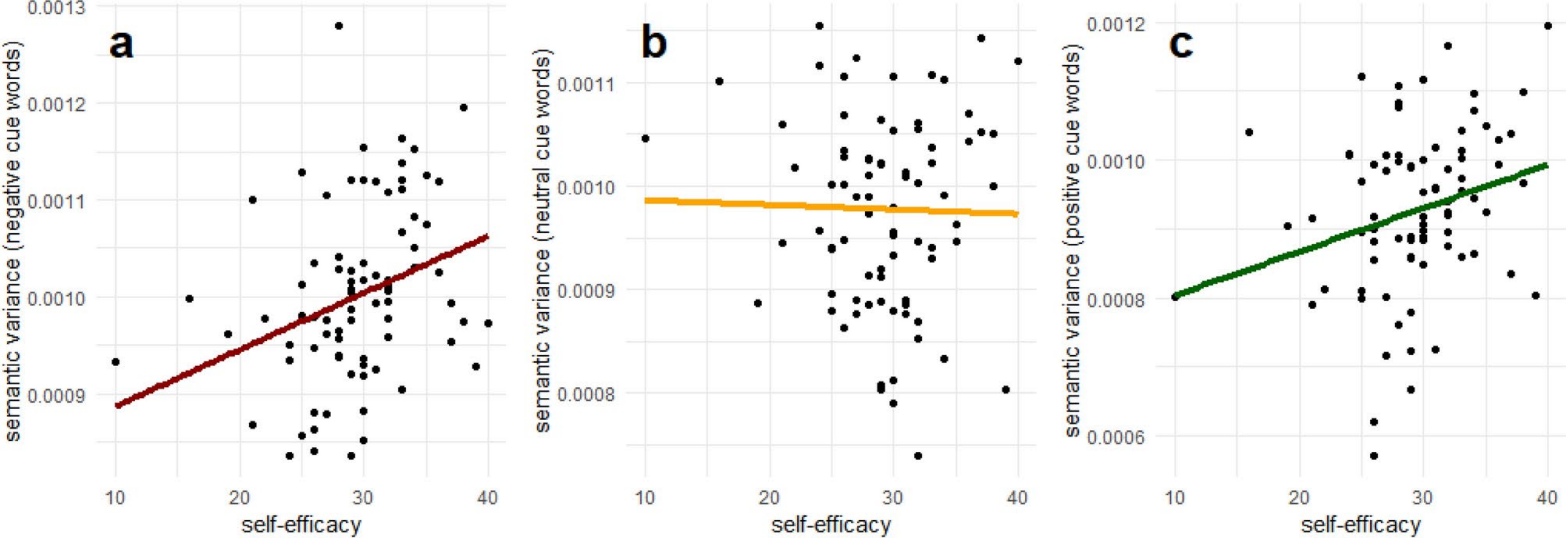
Pearson-correlations between self-efficacy and the semantic variance of mental time travel narratives separately for each content cue word valence. (**a**) There was a positive relation between self-efficacy and the semantic variance across negatively cued, r(80) = 0.32, adj. p = 0.011, and (**c**) positively cued, r(80) = 0.26, adj. p = 0.037, narratives. (**b**) There was no significant correlation between self-efficacy and the semantic variance across neutrally cued narratives, r(80) = − 0.02, adj. p = 0.8. P-values were adjusted using the Holm-Bonferroni method.

The correlation between SE and the semantic variance across all 12 narratives was not significant, r(80) = 0.15, p = 0.2. Subsequently, we examined whether the relationship between the narratives’ semantic variance and SE was dependent on the stimulus valence. p-values were adjusted using the Holm-Bonferroni method. There was a positive relation between SE and the semantic variance across negatively cued, r(80) = 0.32, adj. p = 0.011, and positively cued, r(80) = 0.26, adj. p = 0.037, narratives. There was no significant correlation between SE and the semantic variance across neutrally cued narratives, r(80) = − 0.02, adj. p = 0.8. The correlations are shown in Fig. 3.

### Future-past similarity

To test the assumption that subjects would generally refer to more similar past and future events when these were prompted by the same cue word, we calculated the average future-past similarity between cue word incongruent MTT pairs (e.g., the semantic similarity between the narratives prompted by “anxiety past” and “dispute future”) and compared it to the future-past similarity of congruent cue word pairs (e.g., “anxiety past” and “anxiety future”). A one-sided paired sample t-test confirmed that the future past similarity was higher for congruent [M = 0.4, SD = 0.08] than for incongruent [M = 0.26, SD = 0.05] cue word pairs, t(81) = 17.65, p < 0.001, d = 2.16. Thus, the content of imagined and remembered experiences tended to be related.

To ascertain whether SE might influence the similarity between past and future episodes, we conducted a two-way ANCOVA with within factor *Valence* and covariate *Self-efficacy*. No main effect of the covariate *Self-efficacy* emerged. There was a significant negative correlation between SE and the future-past similarity across all cue words, r(80) = -0.26, p = 0.02. As subjects with higher SE produced less similar narratives, we aimed to determine whether the effect of SE on the future-past similarity would remain even after controlling for the total semantic variance. A multiple regression now only showed a trend toward significance for the predictor SE, β = -0.003, t = -1.9, p = 0.061, η^2^p = 0.04.

## Discussion

The present study introduced novel methods to study the MTT in subjects with varying levels of SE. Subjects provided written narratives describing anticipated and past personal experiences. Based on the literature on SE and prior research describing NLP-derived memory features, we extracted four MTT features which we hypothesized to be related to SE. These measures were first validated using experimental and ML-driven methods. The employed methods were determined to be valid and could be used to study the content of MTT narratives. Our main analyses suggested that high SE coincides with central changes to the (re-)construction of personal episodes. The content of emotional episodes of self-efficacious subjects was less similar, which was evidenced by a higher semantic variance of narrative embeddings in semantic space. With respect to the phenomenological characteristics, subjects with high levels of SE tended to produce more positive narratives in which they assumed a more active role.

The present investigation builds on recent developments in memory research which has seen an increase in the use of NLP models^27,29–31^. We extend these studies by proposing novel textual memory features which may be applied to the study of SE, but also other research domains. In the past, most memory research was limited to methods of phenomenological inquiry and scoring by human raters. These methods have been criticized due to their subjectivity^28^, time consumption^53^ and their inability to capture more abstract textual features that a human rater may not be able to grasp^28^. Consequently, we applied computational methods to investigate the relation between MTT and SE which has been noticeably understudied in this respect. Our findings of an increased semantic variance in self-efficacious subjects align well with previous research showing a negative link between SE and inflexible thinking styles such as worry and rumination^19–22^. Worry and rumination reflect a tendency to repeatedly revisit upsetting personal experiences^54^. As such, they may be conceptualized as inflexible MTT. Thus, MTT flexibility may function as a behavioral measure of perseveration. Future research should investigate the benefits of being able to imagine and remember a semantically diverse set of personal episodes.

With respect to the narratives’ emotionality, our results show that subjects with high SE rated the imagined and remembered experiences as more positive. Interestingly, however, this increased positivity could not be detected when we applied a sentiment analysis to the texts. This is in contrast to previous research which demonstrated an increase use of positive words after experimental SE induction^14^. There are multiple explanations for this discrepancy. Firstly, sentiment analysis takes into account the entirety of a narrative instead of counting the frequency of positive words as was done in Brown et al.’s study^14^. Because a narrative’s emotionality extends beyond the frequency of positive words, the herein used computational measure of a narrative’s emotionality may be more accurate. Secondly, while we studied natural variations in trait SE, Brown and colleagues utilized an experimental SE induction. It is unclear whether the experimental induction of SE coincides with an unwanted change of affect that might confound the results. Although evidence on the emotionality of remembered experiences in high SE subjects is still sparse, our findings are generally in line with the results of Paersch et al.^55^, who found that high SE causes a reappraisal of negative experiences. We followed a different methodological approach, but found that SE was related to a larger discrepancy between the objective and subjective emotional valence of an experience. Consequently, a compelling explanation of our findings is that self-efficacious subjects remember experiences as more positive irrespective of their objective affective coloring. Future research should explore the functional implications of such reappraisal processes for successful emotional regulation^37^.

High SE coincided with assuming a more active role within the remembered and imagined scenarios. This is in line with the proposition that high SE subjects have a different view of themselves in relation to the world and arising adversities in that they view themselves not as passive bystanders, but as active agents that possess the ability to exert influence on their personal situation^17^. This percept appears to spill over into remembered and imagined personal experiences. SE is often experimentally induced via the remembrance of mastery experiences. Mastery experiences are personal episodes in which a person overcame an obstacle on their own account. In these studies participants are often asked to remember positive episodes that were marked by success vividly and in detail^16,38,56,57^. Thus far, it was not clear whether this kind of MTT is conducive to the enhancement of SE beliefs. Our findings provide support for the idea and common practice that the recalled episodes should be perceived as positive. However, the results of our study also suggest that subjects should retrieve episodes in which they assumed an active role. In the future, it should be determined whether the recall of these experiences induces a superior level of SE.

The most pronounced limitation of this study is its cross-sectional design, which does not allow for causal inference. As MTT has been found to modulate SE^16,57^, and vice versa^14,15,38^, it is unclear whether the MTT characteristics discovered here are a consequence or a cause of increased SE. Additionally, it is unclear whether a third, unconsidered, variable might mediate the observed relationships. Future studies could employ an experimental manipulation of SE (through means other than remembering mastery experiences, such as false feedback [e.g. Refs.^57,58^]) in combination with the herein presented methodology for MTT assessment to determine the existence of a causal relationship. Additionally, one could manipulate participants’ MTT characteristics through specifically designed training protocols (e.g.Ref.^59^) and determine whether this would cause an increase in their SE.

Further, this study did not consider the specificity of MTT narratives, which has been related to a variety of mental health constructs^10,12,38^. There is evidence that both the characteristics and effects of specific vs. unspecific memory recall differ^60,61^. Consequently, future studies should investigate whether the effects we here observed may be dependent on the specificity of MTT.

Another limitations pertains to the recruited sample, which is not representative of the general population. It should be attempted to replicate these results in a demographically more diverse sample.

## Conclusion

In the present study we introduced novel methods of text analysis from the field of NLP to study the content and emotionality of MTT narratives. Combining these with methods of phenomenological inquiry, we find changes to the (re-)construction of personal experiences typical of subjects with a pronounced sense of SE.

## Supplementary Information

Supplementary Information.

## Data Availability

Materials, analysis code and processed data are available from the corresponding author upon reasonable request. The raw data cannot be shared due to data protection concerns.
