# Supplementary material for: Natural language processing reveals differences in mental time travel at higher levels of self-efficacy
Source: Sci Rep. 2024 Oct 25;14:25342. doi: 10.1038/s41598-024-76959-w (PMC11512007; doi:10.1038/s41598-024-76959-w)
Supplement: Supplementary file 1 — Supplementary Information. [file 41598_2024_76959_MOESM1_ESM.docx]

**Supplementary information for: Natural language processing reveals differences in mental time travel at higher levels of self-efficacy**

Laurin Plank and Armin Zlomuzica

**Cue word selection**

Cue words were selected from the Berlin Affective Word List (Reloaded, BAWL-R), in which German words were ranked on dimensions such as emotional valence, arousal and imaginability based on Likert scales^1^. In this word-library, the emotional valence of the words is ranked on a scale ranging from -3 (very negative) through 0 (neutral) to +3 (very positive). The arousal level of the words range from 1 (low) to 5 (high) and the variation of the level of imaginability ranges from 1 (low) to 7 (high). We have selected keywords with a negative emotional valence of ≤ -2, neutral valence = 0, and positive valence of ≥ 2. All selected keywords had a moderate level of imaginability ≥ 3. Compared to negative and positive keywords, neutral keywords were associated with a low level of arousal, while the arousal level induced by both positive and negative keywords was similar.

Only common and frequently used words were selected. The negative cue words were anxiety (German: “Angst”) and dispute (“Streit”). The neutral cue words were carton/box (“Karton”) and screw (“Schraube”). The positive cue words were joy (“Freude”) and love (“Liebe”). The main characteristics of the cue words selected from the BAWL-R are summarized in table S1.

**Table S1**

| Cue word (English translation) | Emotional valence [SD] | Imaginability [SD] | Arousal [SD] | Frequency of usage/mio |
| --- | --- | --- | --- | --- |
| Angst (anxiety) | -2.6[0.69] | 3.89[1.36] | 4.38[0.59] | 101.33 |
| Streit (dispute) | -2.2[0.63] | 4.67[1.8] | 4.05[0.74] | 42.5 |
| Karton (carton/box) | 0[0.97] | 5.09[1.34] | 1.84[0.9] | 6.17 |
| Schraube (screw) | 0[0.69] | 5.91[1.27] | 1.86[0.89] | 4.67 |
| Freude (joy) | 2.7[0.57] | 4.27[1.55] | 3.41[1.33] | 86.33 |
| Liebe (love) | 2.9[0.31] | 3.73[2.14] | 3.63[1.61] | 113.5 |

Main characteristics of the cue words selected from the BAWL-R^1^. Emotional valence was rated on a 7-point Likert scale ranging from -3 (very negative) through 0 (neutral) to +3 (very positive). Imaginability was rated on a 7-point Likert scale ranging from 1 (low) to 7 (high). Arousal was rated on a 5-point Likert scale from 1 (low) to 5 (high). Usage frequency is indicated in usage frequency per million.

**Supplementary results**

**Table S2**

| Dependent variable | Effect | F-statistic | p-value | η²p |
| --- | --- | --- | --- | --- |
| Phenomenological characteristics |  |  |  |  |
| Temporal distance | *Directedness* | 15.93 | <.001 | .16 |
|  | *Directedness* x *Valence* | 4.67 | .011 | .05 |
| Vividness | *Directedness* | 46.76 | <.001 | .36 |
|  | *Valence* | 15.33 | <.001 | .15 |
| Emotional arousal | *Valence* | 72.83 | <.001 | .46 |
| Personal relevance | *Valence* | 52.05 | <.001 | .38 |
| Use of mental imagery | *Valence* | 11.98 | <.001 | .13 |
|  | *Directedness* x *Valence* | 4.36 | .014 | .05 |
| Perspective | *Directedness* | 14.92 | <.001 | .15 |
|  | *Valence* | 4.35 | 0.14 | .05 |
| Active vs. passive role | *Valence* | 3.07 | .049 | .04 |
| Dynamic vs. static | *Valence* | 8.1 | <.001 | .09 |
| Detailedness | *Directedness* | 20.61 | <.001 | .2 |
|  | *Valence* | 11.66 | <.001 | .12 |
|  | *Directedness x Valence* | 3.4 | .036 | .04 |
| Emotional valence | *Directedness* | 11.97 | <.001 | .13 |
|  | *Valence* | 147.15 | <.001 | .64 |
| Text analysis |  |  |  |  |
| Word count | *Valence* | 8.77 | <.001 | .1 |
| Emotional valence (sentiment analysis) | *Valence* | 52.43 | <.001 | .47 |
| Narrative coherence | *Valence* | 7.03 | <.01 | .13 |
|  | *Directedness* x *Valence* | 3.67 | .029 | .07 |

Positive main and interaction effects for the factors *Directedness* and *Valence*. Effect sizes are computed as partial eta squared (η²p).

**Table S3**

| Dependent variable | *Directedness* | *Valence* | Mean[SD] |
| --- | --- | --- | --- |
| Temporal distance |  |  |  |
|  | Past | Negative | 4.01[1.22] |
|  | Future | Negative | 3.4[1.11] |
|  | Past | Neutral | 3.93[1.36] |
|  | Future | Neutral | 3.39[1.28] |
|  | Past | Positive | 3.88[1.26] |
|  | Future | Positive | 3.64[1.15] |
| Vividness |  |  |  |
|  | Past | Negative | 5.11[1.14] |
|  | Future | Negative | 4.45[1.24] |
|  | Past | Neutral | 4.49[1.28] |
|  | Future | Neutral | 4.21[1.28] |
|  | Past | Positive | 5.28[1.15] |
|  | Future | Positive | 5[1.16] |
| Emotional arousal |  |  |  |
|  | Past | Negative | 5.2[1.17] |
|  | Future | Negative | 5.05[1.09] |
|  | Past | Neutral | 3.51[1.25] |
|  | Future | Neutral | 3.76[1.51] |
|  | Past | Positive | 5.31[1.04] |
|  | Future | Positive | 5.25[1.08] |
| Personal relevance |  |  |  |
|  | Past | Negative | 4.3[1.45] |
|  | Future | Negative | 4.59[1.24] |
|  | Past | Neutral | 3.44[1.49] |
|  | Future | Neutral | 3.87[1.53] |
|  | Past | Positive | 5.16[1.1] |
|  | Future | Positive | 5.31[1.32] |
| Use of mental imagery |  |  |  |
|  | Past | Negative | 4.5[1.28] |
|  | Future | Negative | 4.3[1.31] |
|  | Past | Neutral | 4.16[1.34] |
|  | Future | Neutral | 4.57[1.32] |
|  | Past | Positive | 4.68[1.37] |
|  | Future | Positive | 5.07[1.19] |
| Perspective |  |  |  |
|  | Past | Negative | 8.32[2.52] |
|  | Future | Negative | 7.68[2.82] |
|  | Past | Neutral | 8.2[2.39] |
|  | Future | Neutral | 7.6[2.77] |
|  | Past | Positive | 8.78[2.22] |
|  | Future | Positive | 8.38[2.39] |
| Active vs. passive role |  |  |  |
|  | Past | Negative | 4.57[1.27] |
|  | Future | Negative | 4.56[1.32] |
|  | Past | Neutral | 4.77[1.31] |
|  | Future | Neutral | 5.06[1.21] |
|  | Past | Positive | 4.86[1.21] |
|  | Future | Positive | 5.05[1.28] |
| Dynamic vs. static |  |  |  |
|  | Past | Negative | 4.39[1.35] |
|  | Future | Negative | 4.17[1.23] |
|  | Past | Neutral | 4.33[1.17] |
|  | Future | Neutral | 4.20[1.18] |
|  | Past | Positive | 4.61[1.16] |
|  | Future | Positive | 4.81[1.19] |
| Detailedness |  |  |  |
|  | Past | Negative | 4.72[1.16] |
|  | Future | Negative | 3.95[1.31] |
|  | Past | Neutral | 4.33[1.24] |
|  | Future | Neutral | 4.17[1.23] |
|  | Past | Positive | 4.94[1.18] |
|  | Future | Positive | 4.71[1.15] |
| Emotional valence |  |  |  |
|  | Past | Negative | 2.44[1.17] |
|  | Future | Negative | 2.74[1.03] |
|  | Past | Neutral | 4.37[1.16] |
|  | Future | Neutral | 4.75[1.19] |
|  | Past | Positive | 5.54[1.29] |
|  | Future | Positive | 6.07[1.03] |
| Word count |  |  |  |
|  | Past | Negative | 70.57[31.29] |
|  | Future | Negative | 61.49[26.96] |
|  | Past | Neutral | 62.12[28.27] |
|  | Future | Neutral | 59.75[27.57] |
|  | Past | Positive | 67.7[28.5] |
|  | Future | Positive | 66.72[28.54] |
| Emotional valence (sentiment analysis) |  |  |  |
|  | Past | Negative | -0.02[0.21] |
|  | Future | Negative | -0.01[0.23] |
|  | Past | Neutral | 0.08[0.18] |
|  | Future | Neutral | 0.07[0.19] |
|  | Past | Positive | 0.28[0.24] |
|  | Future | Positive | 0.29[0.19] |
| Narrative coherence |  |  |  |
|  | Past | Negative | 0.23[0.08] |
|  | Future | Negative | 0.24[0.07] |
|  | Past | Neutral | 0.23[0.09] |
|  | Future | Neutral | 0.23[0.08] |
|  | Past | Positive | 0.21[0.06] |
|  | Future | Positive | 0.24[0.07] |
| Future-past similarity |  |  |  |
|  |  | Negative | 0.37[0.1] |
|  |  | Neutral | 0.42[0.11] |
|  |  | Positive | 0.38[0.1] |
| Semantic variance |  |  |  |
|  | Total* | | 0.0013[0.000086] |
|  | Past |  | 0.0012[0.000088] |
|  | Future |  | 0.0011[0.000098] |
|  |  | Negative | 0.001[0.000092] |
|  |  | Neutral | 0.00098[0.000094] |
|  |  | Positive | 0.00093[0.00012] |

Descriptive statistics for all dependent variables across all factor levels of *Directedness* and *Valence*. *Only in the case of semantic variance total scores do not reflect the average of sub scores. This is because semantic variance is a spatial measure reflecting the relationship between trials, not an aggregate of trial-level scores.

***Post-hoc tests for the factors Directedness and Valence***

As a post-hoc test for the factors, we used Tukey’s Honestly Significant Difference (HSD) tests.

***Temporal distance from the present***. Only for negative (p<.01) and neutral cues (p=.016), past MTTs were temporally more distant than future MTTs.

***Vividness.*** Only for future-oriented trials, positively cued MTTs were more vivid than negatively cued MTTs (p=.012). For both past-oriented (p<.001) and future-oriented (p<.001) trials, positively cued MTTs were more vivid than neutrally cued MTTs. Only for past-oriented trials, negatively cued MTTs were more vivid than neutrally cued MTTs (p<.01). Lastly, only for negatively cued trials, past-oriented MTTs were more vivid than future-oriented MTTs (p<.001).

***Emotional arousal.*** For both past-oriented (p<.001) and future-oriented (p<.001) trials, positively cued MTTs were more arousing than neutrally cued MTTs. For both past-oriented (p<.001) and future-oriented (p<.001) trials, negatively cued MTTs were more arousing than neutrally cued MTTs.

***Personal relevance.*** For both past-oriented (p<.001) and future-oriented (p<.01) trials, positively cued MTTs were more relevant than negatively cued MTTs***.*** For both past-oriented (p<.001) and future-oriented (p<.001) trials, positively cued MTTs were more relevant than neutrally cued MTTs. For both past-oriented (p<.001) and future-oriented (p=.002) trials, negatively cued MTTs were more relevant than neutrally cued MTTs.

***Use of mental imagery.*** Use of mental imagery was greater for positively cued MTTs than for negatively cued MTTs, but only in future-oriented trials (p<.001). Additionally, for past-oriented trials, use of mental imagery was greater for positively cued MTTs than for neutrally cued MTTs (p=.041).

***Perspective (field vs. observer).*** There were no significant meaningful group differences.

***Active vs. passive role.*** Only for future-oriented trials, participants reported a more active role for neutrally cued MTTs than for negatively cued MTTs (p=.041).

***Dynamic vs. static scenes.*** Only for future-oriented trials, positively cued MTTs were more dynamic than negatively cued MTTs (p=.001) and neutrally cued MTTs (p<.01).

***Detailedness.*** For both future (p=.015) and past-oriented (p<.01) trials, positively cued MTTs were more detailed than neutrally cued MTTs. Furthermore, only for the future-oriented trials, positively cued MTTs were more detailed than negatively cued MTTs (p<.001). Additionally, only for negatively cued trials, past-oriented MTTs were more detailed than future-oriented MTTs (p<.001).

***Emotional valence.*** For both past-oriented (p<.001) and future-oriented trials (p<.001), positively cued MTTs were more positive than negatively cued MTTs. For both past-oriented (p<.001) and future-oriented (p<.001) trials, positively cued MTTs were more positive than neutrally cued MTTs. For both past-oriented (p<.001) and future-oriented (p<.001) trials, neutrally cued MTTs were more positive than negatively cued MTTs. Additionally, only for positively cued trials, future-oriented MTTs were more positive than past-oriented MTTs (p<.01).

***Wordcount.*** No significant comparisons emerged.

***Emotional valence (sentiment analysis).*** For both past-oriented (p<.001) and future-oriented (p<.001) trials, positively cued MTTs were more positive than negatively cued MTTs. For both past-oriented (p<.001) and future-oriented (p<.001) trials, positively cued MTTs were more positive than neutrally cued MTTs (p<.001). Only for past-oriented trials, neutrally cued MTTs were more positive than negatively cued MTTs (p=.017).

**Future-past similarity**. The future-past similarity for the negative (p<.01) and for the positive cue words (p=.021) was lower than that for the neutral cue words.

***Narrative coherence***. No significant comparisons emerged.

**References**

1. Võ, M. L. *et al.* The Berlin affective word list reloaded (BAWL-R). *Behav. Res. Methods* **41**, 534–538 (2009).
